# Improved glycaemic variability and basal insulin dose reduction during a running competition in recreationally active adults with type 1 diabetes—A single-centre, prospective, controlled observational study

**DOI:** 10.1371/journal.pone.0239091

**Published:** 2020-09-11

**Authors:** Othmar Moser, Alexander Mueller, Max L. Eckstein, Haris Ziko, Felix Aberer, Gerlies Treiber, Christina Unteregger, Harald Kojzar, Julia K. Mader, Caren Sourij, Peter Pferschy, Anna Obermayer, Norbert Tripolt, Harald Sourij

**Affiliations:** 1 Division of Endocrinology and Diabetology, Department of Internal Medicine, Medical University of Graz, Graz, Austria; 2 Exercise Physiology, Training & Training Therapy Research Group, Institute of Sports Science, University of Graz, Graz, Austria; Universidad de Cantabria - University Hospital Marqués de Valdecilla, SPAIN

## Abstract

**Introduction:**

To investigate the glycaemic response, macronutrient intake and insulin management in people with type 1 diabetes (T1D) compared to healthy individuals around a running competition.

**Material and methods:**

This was a single-centre, prospective, controlled observational study performed in individuals with T1D and healthy people. 24 people (12 T1D) were included in this study (age: T1D 41±12 vs. healthy 38±6 years, females: 3 vs. 6, BMI: 25.53.0 vs. 22.9±2.8 kg/m^2^). Both groups received an intermittently scanned continuous glucose monitoring (isCGM; FreeStyle Libre 1, Abbott, USA) system to assess glycaemia 24 hours before, during and 24 hours after a running competition. During this period, participants recorded their food intake and insulin administration. Data were analysed via ANOVA and mixed model analyses with post-hoc testing (p≤0.05).

**Results:**

For overall glycaemic ranges in comparison of groups, significant differences were found for time in range (T1D 63±21% vs. healthy 89±13%, p = 0.001), time above range (TAR) 1 (T1D 21±15% vs. healthy 0±0%, p<0.001) and TAR 2 (T1D 8 [0–16%] vs. healthy 0±0%, p<0.001). When glycaemic variability was assessed, people with T1D had a higher glycaemic variability compared to healthy individuals (p<0.0001). Basal insulin dose was significantly reduced when compared against the regular pre-study basal insulin dose (pre-study 22±6 vs. pre-competition day 11±9 (-50±41%), p = 0.02; competition day 15±5 (-32± 1%)).

**Conclusion:**

People with T1D have impaired glucose responses around a running competition compared to healthy individuals. However, basal insulin dose reductions were sufficient to prevent further dysglycaemia.

**Clinical trial ID:**

drks.de; DRKS00019886

## Introduction

For adults with and without type 1 diabetes (T1D), 150 minutes per week of moderate-intensity exercise or 75 minutes per week of intense exercise with no more than two days of rest are recommended [1,2]. This recommendation is supported by studies showing that a more physically active lifestyle is associated with a lower mortality rate [3], improved glycaemic control and a reduced risk of retinopathy, microalbuminuria, hypertension as well as dyslipidaemia [4]. Since exercise-induced hypoglycaemia is a major complication of physical activity and exercise in people with T1D [4,5], only ~45% achieve current recommendations [6,7] and this might be based on fear of losing glycaemic control [8].

In contrast to this elevated risk of dysglycaemia, more people with T1D participate in extreme sports competitions and hence prove that T1D *per se* is not a reason for being physically inactive [9]. In several observational studies it was shown that people with T1D can participate in sports like running competitions [10–12], Ironman [13] or 7-day stage professional cycling races [14]. For running a half marathon, it was shown that recreationally active people with T1D reduced their total daily insulin dose by 14–18% [10]; especially for the pre-competition breakfast, bolus insulin dose was reduced by 15–32% resulting in blood glucose increase of 62–108 mg/dL [10]. However, these data were only assessed by means of capillary blood glucose monitoring (SMBG), not providing a complete glucose profile as seen for continuous glucose monitoring (CGM) systems. A recent observational study in professional cyclists with T1D using CGM systems showed that the athletes had good in-race glycaemia, but were prone to hypoglycaemia during post-exercise nighttime period. Similar results were shown in a larger group of professional cyclists with T1D, detailing also a greater risk of nocturnal hypoglycaemia during a training camp [15]. However, as already seen in recreationally active people with T1D, performing an evening-exercise session increases the risk of late-onset nocturnal hypoglycaemia [15,16]. Increased protein levels of insulin independent glucose transporter type 4 (GLUT-4) might be the main trigger for exercise-induced hypoglycaemia [17]. Intriguingly, some studies also reported exercise-induced hypoglycaemia and low glucose levels in people not having diabetes [18–20], but without known pathologic background. While athletes with T1D are used to regular training and competition and are familiar with the required therapy adjustments, however, the question arises how recreationally active people with T1D who engage more and more in sports competitions are managing their therapy around a competition.

Therefore, the aim of the present study was to investigate glycaemic responses, macronutrient intake and insulin management in people with T1D and healthy individuals before, during and after a running competition.

## Material and methods

This was a single-centre, prospective, controlled observational study performed in individuals with T1D and healthy people. For this study, participants were recruited from August 2019 until September 2019 by advertisement in local newspapers.

### Eligibility criteria

For both groups, people aged 18–65 years (both inclusive) with a body mass index of 18.5–30.0 kg/m^2^ (both inclusive) who planned to participate in the *Graz Marathon* (AT) (distance 10.0 or 21.1 km) were eligible for this study. Participants with T1D were eligible if glycated haemoglobin A_1c_ (HbA_1c_) was 5.5–10.0% (37–113 mmol/mol) without a diagnosis of a relevant comorbidity and using an intermittently scanned CGM (isCGM) system in daily routine. The control group had to be without a diagnosis of a relevant comorbidity and was assessed by means of a general medical check-up. None of the participants of the healthy control group had any comorbidity or any sign of an impaired glucose metabolism as assessed by means of isCGM data.

### Screening visit

The screening visit took place at least one week prior to the running competition. A venous blood sample was taken for the assessment of HbA_1c_ in the group of individuals with T1D. A medical assessment was performed for both groups to ensure cardio-vascular health. Groups received a food diary to record food intake (type, amount and time) and type, dose and time of insulin administration (T1D group) that was compared against the isCGM trace. Participants were asked to record the type, intensity and duration of physical activity and exercise performed for the investigational period 24 hrs prior to the competition until 24 hrs after the competition. Additionally, participants with T1D received standard recommendations on how to adapt therapy around the running competition based on previous position and consensus statements but they did not adhere to a specific protocol [1,2]. A few days before the competition healthy participants received an isCGM device and the sensor was inserted by the research team to monitor their glucose levels.

### Trial visit

One hour prior to the start of the running competition, participants received a heart rate monitor (S810, Polar, Finland), to assess the heart rate during the running competition in relation to Fox's 220-age-predicted maximum heart rate [21]. Additionally, in the group of T1D, a pre-competition capillary blood glucose measurement was performed for safety reasons. At least every 4–5 km, a medically trained researcher was available for the safety of the participants.

### Statistical analyses

Sensor glucose data obtained from isCGM were stratified for time below range level 2 (TBR 2; <54 mg/dL [<3.0 mmol/L]), time below range level 1 (TBR 1; 54–69 mg/dL [3.0–3.8 mmol/L]), time in range (TIR; 70–180 mg/dL [3.9–10.0 mmol/L]), time above range level 1 (TAR 1; >180–250 mg/dL [>10.0–13.9 mmol/L]) and time above range level 2 (TAR 2; >250 mg/dL [>13.9 mmol/L]) [22]. Data were also stratified for the 24 hrs-pre-competition, competition and 24 hrs-post-competition phases and further analysed for daytime (06.00 A.M–11.59 P.M) and nighttime periods (12.00 A.M–05.59 A.M). Additionally, for all phases and periods median sensor glucose and markers of glycaemic variability (and coefficient of variation) in sensor glucose were investigated. Data were assessed for distribution by means of Shapiro-Wilk test. Values are given as mean ± SD or median [interquartile range]. Participants’ characteristics, heart rate, distance, and duration of the running competition as well as overall glycaemia were compared by means of unpaired t-test between groups. Within-group-glycaemia for the pre-, during and post-competition phase was analysed via Friedman-test and Dunn's post-hoc test. Groups were compared via 2-way ANOVA and Sidak's post-hoc test (p<0.05). All analyses followed an intention to treat manner. A prospective sample size estimation was not performed due the nature of the running competition. A post-hoc power analysis was performed to compute the achieved power for glycaemia between the two groups.

### Ethical consideration

The study protocol was approved by the local ethics committee of the Medical University of Graz (AUT) (30–462 ex 17/18) and registered at the German Clinical Trials Register (drks.de; DRKS00019886). This study was conducted in full conformity with the 1964 declaration of Helsinki and all subsequent revisions as well as in accordance with the guidelines laid down by the International Conference on Harmonisation for Good Clinical Practice (ICH GCP E6 guidelines).

## Results

**Fig 1:**
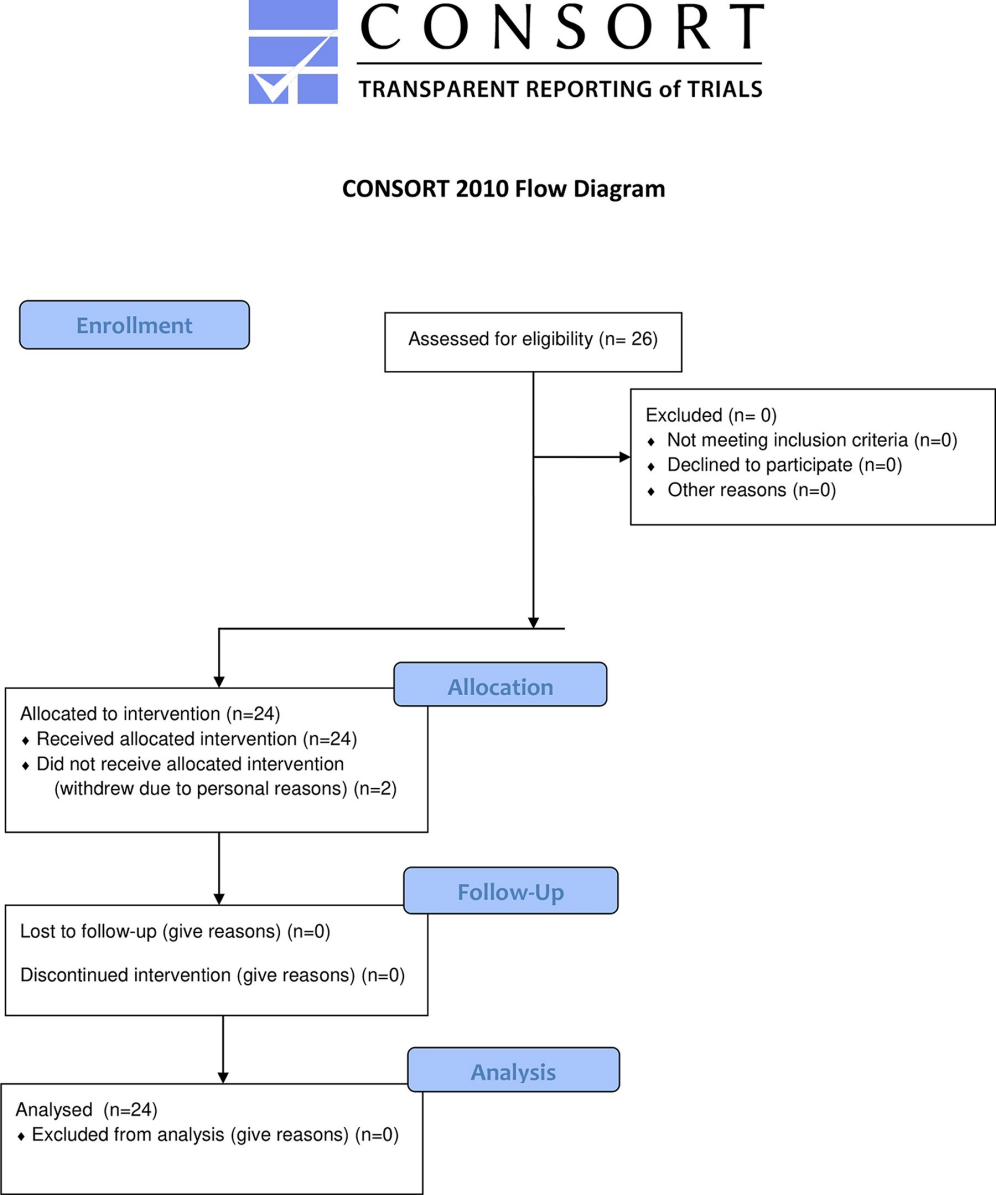
Consort flow diagram.

Out of 26 people screened, two people with T1D withdrew consent to participate due to personal reasons, resulting in a total of 24 participants (12 per group) (Table 1, Fig 1). During the pre-, during and post-competition phases, no episode of severe hypoglycaemia requiring external assistance occurred. All isCGM sensor glucose and heart rate data were available without any data loss during the three-day period. Seven people were using continuous subcutaneous insulin infusion (CSII) therapy (5 insulin Aspart, 2 insulin Lispro) and 5 people were using multiple daily insulin injection (MDI) therapy (basal insulin: 4 insulin Degludec, 1 insulin Glargine U-300; bolus insulin: 5 insulin Aspart).

**Table 1:** Participants’ characteristics in comparison of groups T1D and healthy.

	T1D	Healthy controls	p-value
**Age (years)**	41 ± 12	38 ± 6	0.39
**BMI (kg/m**^**2**^**)**	25.5 ± 3.0	22.9 ± 2.8	0.05
**Gender (F/M)**	3/9	6/6	N/A
**eA**_**1c**_ **(% [mmol/mol])**	7.5 ± 1.5 [58 ± 17]	4.6 ± 0.3 [26 ± 9]	<0.0001
**Diabetes duration (years)**	12 ± 10	-	-
**TD basal dose (I.U)**	22 ± 6	-	-
**TD bolus dose (I.U)**	18 ± 4	-	-
**TDD (I.U)**	37 ± 14	-	-

BMI = body mass index; eA_1c_ = estimated A_1c_ from isCGM (last week prior to competition); TD = total daily; TDD = total daily dose; MDI = multiple daily injections; CSII = continuous subcutaneous insulin infusion.

### Exercise performance data

All of the participants finished their scheduled run distances. In the group of participants with T1D, 5 people ran 21.2 km and 7 people ran 10 km; in the group of healthy individuals, 6 people ran 21.2 km and 6 people ran 10 km. Total competition time and competition distance was not significantly different in comparison of groups (time: T1D 85 ± 29 min vs. healthy 89 ± 37 min, p = 0.81; distance: T1D 14.0 ± 5.6 km vs. healthy 15.5 ± 5.7 km, p = 0.53). Absolute (HR_mean_) and relative mean heart rate (%HR_max_) during the competition was similar for both groups (HR_mean_: T1D 172 ± 8 beats/min vs. healthy 169 ± 12 beats/min; %HR_max_: T1D 95 ± 5% vs. healthy 92 ± 4.7%, p = 0.23). The running distance did not significantly alter mean sensor glucose levels for both groups (21.1 km runners 115 ± 46 mg/dL [6.4 ± 2.6 mmol/L] vs. 10 km runners 127 ± 44 mg/dL [7.0 ± 2.4 mmol/L], p = 0.51) and within the group of T1D (p = 0.75) and healthy (p = 0.70).

### Sensor glucose data

#### Glycaemia

Over the entire study period, mean ± SD sensor glucose levels were significantly higher in people with T1D compared to healthy individuals (T1D 148 ± 37 mg/dL [8.3 ± 2.1 mmol/L] vs. 86 ± 7 mg/dL [4.8 ± 0.4 mmol/L], p < 0.001). The achieved power (1-β error probability) for glycaemia in comparison of groups was 0.99 (nocentrality parameter δ 5.70, critical t 1.71, Df 22), representing a sufficient number of participants in comparison of groups.

**Fig 2:**
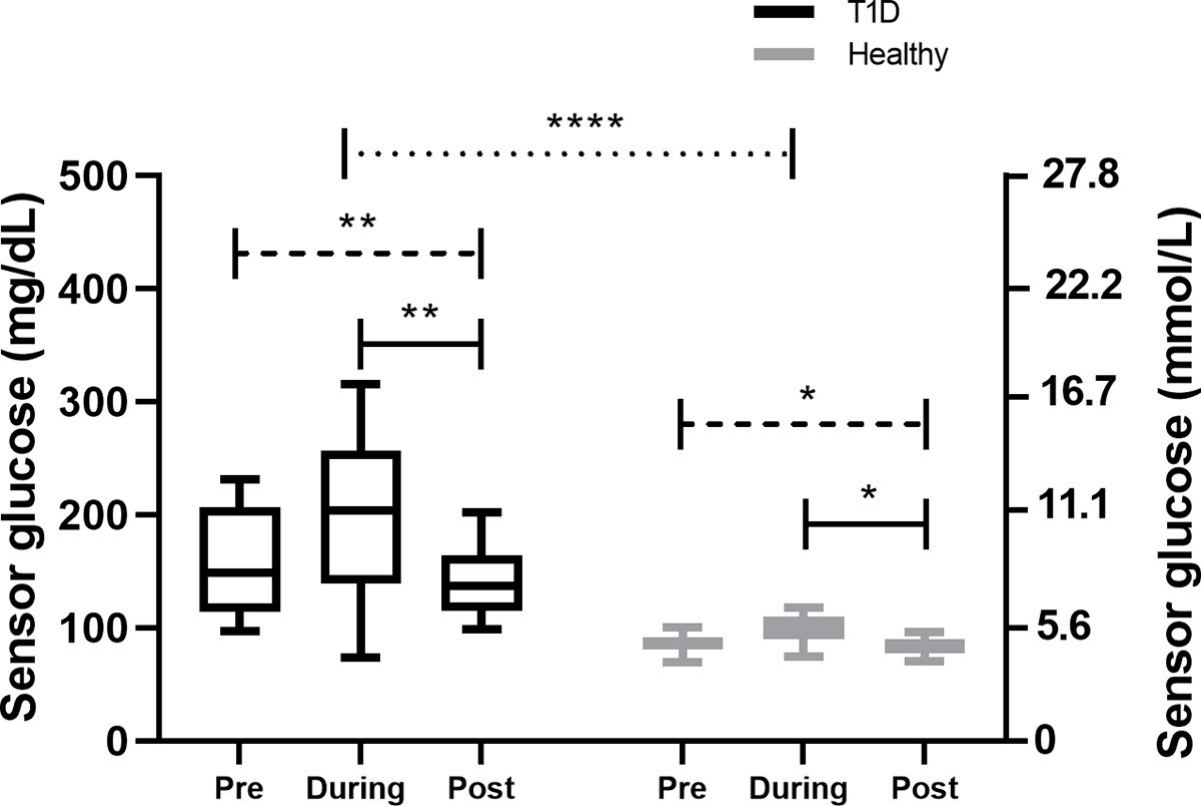
Sensor glucose in comparison of the 24 hrs-pre (Pre), during (During) and 24 hrs-post-competition phase (Post).

Within the T1D group, mean sensor glucose values were significantly different when comparing the 24 hrs-pre, during and 24 hrs-post-competition periods (p = 0.009; Fig 2). Similar results were found for healthy individuals, with significant differences in comparison of these three phases (p = 0.01; Fig 2). Both groups showed similar sensor glucose pattern over the three phases (p = 0.03).

#### Pre-, during and post-competition glycaemic ranges

**Fig 3:**
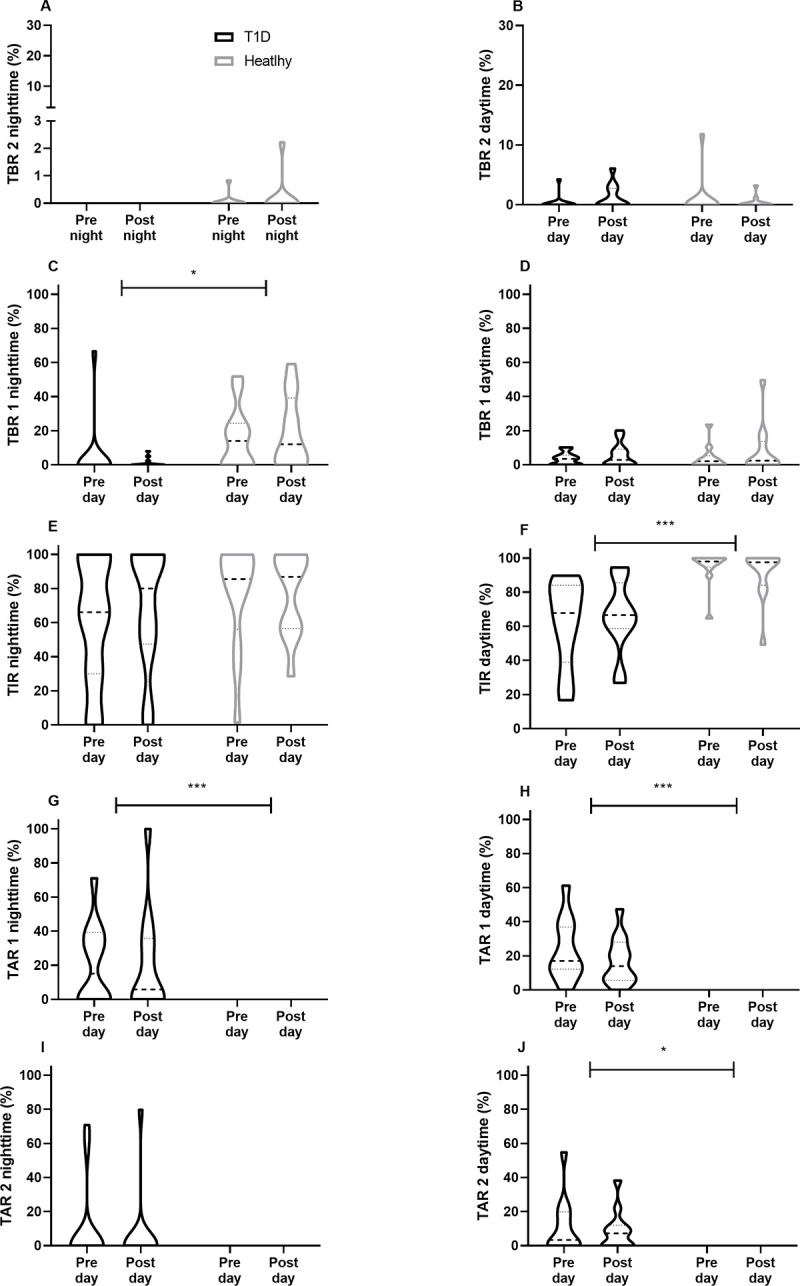
Comparison of pre-competition vs. post-competition glycaemia stratified for nighttime and daytime glycaemia in comparison of individuals with type 1 diabetes (T1D) and healthy individuals.

For overall glycaemic ranges in comparison of groups, significant differences were found for TIR (T1D 63 ± 21% vs. healthy 89 ± 13%, p = 0.001), TAR 1 (T1D 21 ± 15% vs. healthy 0 ± 0%, p < 0.001) and TAR 2 (T1D 8 [0–16%] vs. healthy 0 ± 0%, p < 0.001). No significant differences were found when comparing groups for TBR 2 (T1D 0 [0–1%] vs. healthy 0 [0–2%], p = 0.89) and TBR 1 (T1D 4 [0–8%] vs. healthy 6 [0–16%], p = 0.31) (Fig 3).

Comparison within and between groups for glycaemic ranges stratified for the 24 hrs-pre, during and 24 hrs-post-competition periods is given in Table 2.

**Table 2:** Comparison of glycaemic range defined as time below range level 2 (TBR 2; <54 mg/dL [<3.0 mmol/L]), time below range level 1 (TBR 1; 54–69 mg/dL [3.0–3.8 mmol/L]), time in range (TIR; 70–180 mg/dL [3.9–10.0 mmol/L]), time above range level 1 (TAR 1; >18–250 mg/dL [>10.0–13.9 mmol/L]) and time above range level 2 (TAR 2; >250 mg/dL [>13.9 mmol/L]). Values are given as median [interquartile range].

	T1D			p-value T1D	Healthy			p-value Healthy	p-value T1D versus Healthy
	Pre	During	Post		Pre	During	Post		
**TBR 2 (%)**	0 [0–0]	0 [0–0]	0 [0–2]	0.009	0 [0–0.2]	0 [0–0]	0 [0–2]	0.11	0.34
**TBR 1 (%)**	3 [0–6]	0 [0–0]	2 [0–7]	0.03	5 [0–13]	0 [0–0]	5 [0–22]	0.03	0.22
**TIR (%)**	69 [39–80]	40 [0–60]	72 [10–87]	0.17	95 [87–100]	100 [100–100]	95 [77–100]	0.01	0.0004
**TAR 1 (%)**	22 [9–35]	20 [2–39]	16 [7–23]	0.76	0 [0–0]	0 [0–0]	0 [0–0]	>0.99	0.0004
**TAR 2 (%)**	5 [0–19]	9 [0–55]	6 [0–15]	0.28	0 [0–0]	0 [0–0]	0 [0–0]	>0.99	0.005

#### Pre- versus post-competition nighttime and daytime glycaemic ranges

No significant differences were found in comparison of night vs. daytime glycaemia for both groups (p > 0.05). Comparison within and between groups for nighttime and daytime glycaemia by means of pre- and post-completion is shown in Fig 3.

### Glycaemic variability

**Fig 4:**
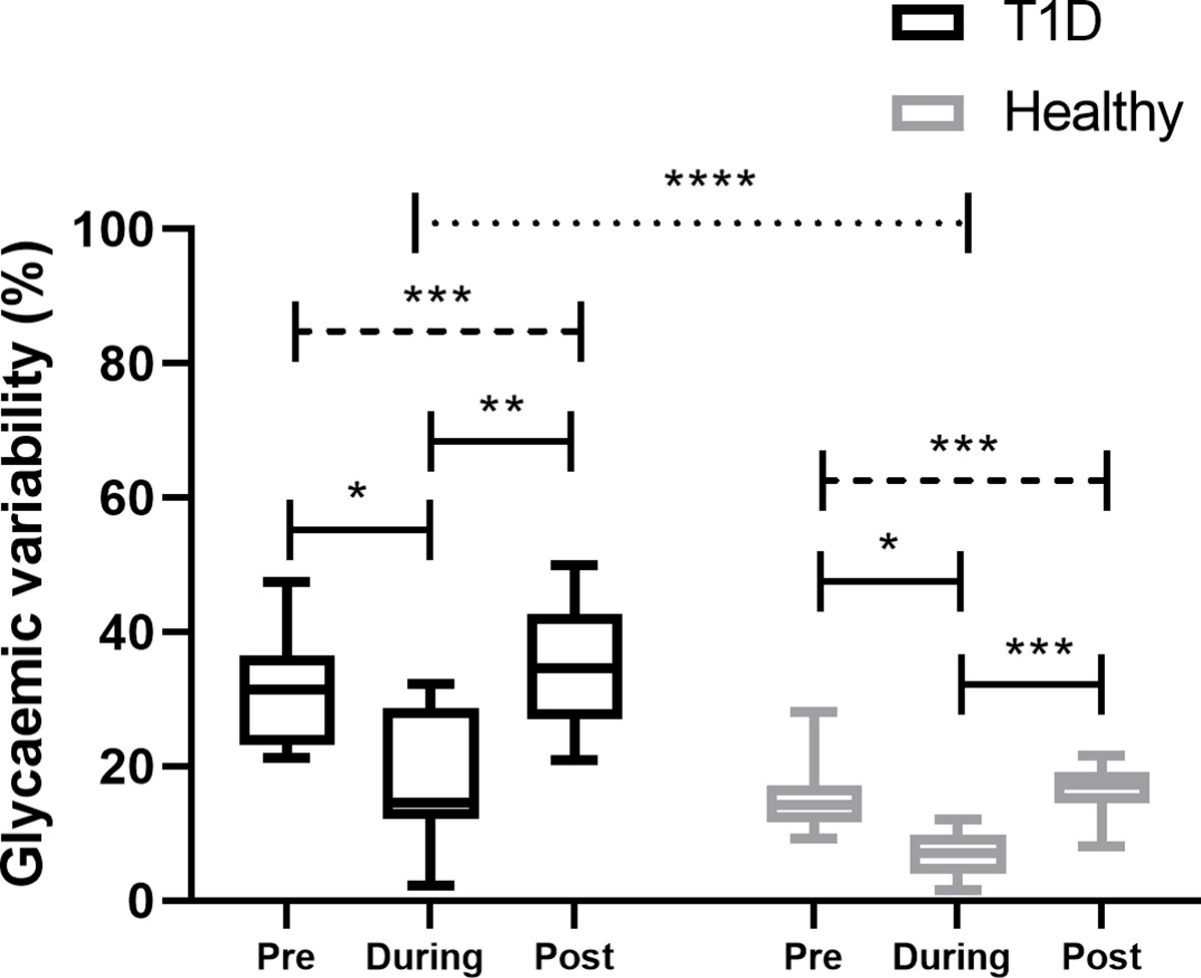
Glycaemic variability assessed via coefficient of variation for the phases pre-, during and post-competition within and in comparison of groups.

When glycaemic variability was assessed via coefficient of variation, overall people with T1D had a higher glycaemic variability compared to healthy individuals (p < 0.0001). Within the group of T1D, overall significant differences were found for coefficient of variation (p = 0.0008), with the lowest glycaemic variability during competition when compared against the pre- (p = 0.02) and post-competition period (p = 0.006). Similar findings were seen for healthy individuals with overall significant differences for coefficient of variation in comparison of the three phases (p = 0.003), showing the lowest glycaemic variability during competition when compared against the pre- (p = 0.01) and post-competition period (p = 0.0003) (Fig 4).

### Macronutrient intake and insulin administration

#### Macronutrient intake

During the competition, both groups consumed similar amounts of macronutrients (carbohydrates: T1D 27g [0–47] vs. healthy 17g [0–28], p > 0.99; protein: T1D 0g [0–0] vs. healthy 0g [0–2], p > 0.99; fat: T1D 0g [0–0] vs. healthy 0g [0–0], p > 0.99). Within the group T1D in comparison of pre- vs. post-competition, no significant differences were found for carbohydrates (pre 187 ± 73g vs. post 164 ± 42g, p = 0.34), protein (pre 57 ± 29g vs. post 57 ± 24g, p = 0.99) and fat (pre 38 ± 32g vs. post 49 ± 29g, p = 0.13). Within the group of healthy individuals in comparison of pre- vs. post-competition, significant differences were found for carbohydrates (pre 235 ± 97g vs. post 172 ± 93g, p = 0.02), protein (pre 81 ± 25g vs. post 58 ± 38g, p = 0.03) and fat (pre 81 ± 38g vs. 55 ± 39g, p = 0.02). In comparison of groups, no significant differences were found for the pre- and post-competition period in regards to carbohydrate intake (p = 0.35), protein (p = 0.28) and fat (p = 0.09).

### Insulin administration

Regular basal insulin dose was in some periods significantly reduced when compared against the usual basal insulin dose during the pre-study period (pre-study 22 ± 6 IU vs. pre-competition day 11 ± 9 IU (-50 ± 41%), p = 0.02; competition day 15 ± 5 IU (-32 ± 11%), p = 0.02; post-competition day 17 ± 10 IU (-23 ± 10%), p = 0.31). However, basal insulin doses on pre-competition day, competition day and post-competition were comparable (p = 0.07). Pre-study daily bolus insulin dose was not significantly reduced when compared against the study period (pre-study 18 ± 4 IU vs. pre-competition day 17 ± 7 IU (-0.6 ± 0.3%), p = 0.66; competition day 16 ± 5 IU (-2.2 ± 0.7%), p = 0.58; post-competition day 20 ± 5 IU (+1 ± 0.25%), p = 0.29). Pre-competition day, competition day and post-competition bolus insulin doses were not significantly different (p = 0.31).

## Discussion

Our study showed that people with T1D have unsurprisingly higher sensor glucose levels around a running competition, however, the glucose pattern in preparation to, during and after a running competition were similar to those seen in healthy individuals. Intriguingly, people with T1D showed a mean sensor glucose of ~200 mg/dL (11.1 mmol/L) during the running competition that is higher than the recommended range of 126–180 mg/dL (7.0–10.0 mmol/L) and to that what was seen in our group of healthy individuals (~100 mg/dL [5.6 mmol/L] [1,2]. These slightly elevated sensor glucose levels during the competition are in line with data from professional cyclists during a race, where ~20─25% of total race time was spent in TAR 1 [14,23] However, in our data, TIR during the competition was found at ~40% of total race time, which is lower than seen in previously published studies in controlled settings for cycling [24], running [25,26] and as observed in our healthy group (~100%). The lower TIR seen in our study might be due to the real life set-up of a running competition, since it is more difficult to manage glucose as tightly as under lab conditions [24–26], hence under lab [24–26] and training conditions [15], TIR in range was found to be between 68–81%. An optimal TIR of >80% during exercise or a competition [27] might only be achieved if glucose levels are monitored tightly (~5 minutely) and respective adjustments are made to either the insulin dosing or the carbohydrate consumption [24]; however, this is not feasible during a running competition. Notable, during the running competition, participants with T1D did not show any TBR 1 or TBR 2 as also seen in the healthy group, which might be due to the safe but elevated mean glucose levels, as also confirmed by a TAR 1 of ~20% and TAR 2 of ~9%. Furthermore, the running competition did not increase the risk of late-onset hypoglycaemia when compared with pre-competition glycaemia that was also confirmed when glycaemia was stratified for the daytime and nighttime period. Since the running competition was started at around 10 A.M, people with T1D were less prone to late-onset hypoglycaemia and nocturnal hypoglycaemia as compared to previous studies [14–16]. Bolus insulin dose was not adapted and carbohydrate intake was unchanged, while the basal insulin dose was reduced by almost 50% on the day before, and on the day of the running competition. Although all of the participants running on MDI were using a second-generation basal insulin analogue, the basal insulin dose was reduced, which is in line with previous studies [28,29]. As seen in previous studies, participants in our study reduced the basal insulin rate for CSII by almost 50% at the competition day, which was sufficient to protect against hypoglycaemia [30–32].

Our study is not without limitations, since we used an isCGM, which is known to be less accurate during exercise and hypoglycaemia [33,34]. However, due to the low glycaemic variability and the associated low rate of glucose change as seen during the running competition and the analysis including 3 days in total, we do expect a negligible influence on the overall results [35].

## Conclusions

To the best of our knowledge, this is the first study assessing sensor glucose levels around a running competition in people with T1D compared to healthy individuals. People with T1D have impaired glucose responses around a running competition when compared against healthy individuals. However, basal insulin dose reductions were sufficient to prevent further dysglycaemia. Taking into account that the participants with T1D showed in general a very low time below range, a running competition can be safely performed in people with T1D, using MDI or CSII as standard therapy. Therefore, our findings should encourage people with T1D to engage regularly in sports.

## Supporting information

S1 Checklist TREND statement checklist. (PDF)

S1 Protocol (PDF)
