# Supplementary material for: Improved glycaemic variability and basal insulin dose reduction during a running competition in recreationally active adults with type 1 diabetes—A single-centre, prospective, controlled observational study
Source: PLoS One. 2020 Sep 11;15(9):e0239091. doi: 10.1371/journal.pone.0239091 (PMC7485886; doi:10.1371/journal.pone.0239091)
Supplement: S1 Protocol — (PDF) [file pone.0239091.s002.pdf]

**Protocol ID**	ExGlyCo
**Title**	Effects of regular physical **ex**ercise in preparation for a road running competition on **gly**caemic **co**ntrol in individuals with type 1 diabetes – a prospective non-interventional study (ExGlyCo)
**Version**	2.1
**Date**	07/10/2019
**Sponsor**	Medical University of Graz<br>Auenbruggerplatz 15<br>8036 Graz, Austria<br>Assoc.Prof. Dr. Harald Sourij<br>E-Mail: ha.sourij@medunigraz.at<br>Tel: +43 316 385 81310<br>Fax: +43 316 385 13428
**Principal Investigator**	**Ass-Prof. Dr. Harald Sourij**<br>Division of Endocrinology and Diabetology<br>Department of Internal Medicine<br>Medical University of Graz<br>Auenbruggerplatz 15<br>8036 Graz, Austria<br>E-Mail: ha.sourij@medunigraz.at<br>Tel: +43 316 385 81310<br>Fax: +43 316 385 13428
**Co-Principal Investigator**	**Dr. Othmar Moser**<br>Swansea University<br>Applied Sport, Technology, Exercise and Medicine Research Centre (A-STEM)<br>A111 Engineering East, Fabian Way, Crymlyn Burrows<br>Swansea University, Swansea, SA1 8EN, UK<br>E-Mail: othmar.moser@swansea.ac.uk<br>Tel: +44 7757 062851
**Investigators**	**Felix Aberer, MD**<br>Medical University of Graz<br>Division of Endocrinology and Diabetology<br>Auenbruggerplatz 15<br>8036 Graz, Austria<br>E-Mail: felix.aberer@medunigraz.at<br>Tel: +43 316 385 86113

Gerd Köhler, MD
Medical University of Graz
Division of Endocrinology and Diabetology
Auenbruggerplatz 15
8036 Graz / Austria
E-Mail: gerd.koehler@klinikum-graz.at
Tel: +43 316 385 82033

Ao. Prof. Mag. Dr. Peter Hofmann
University of Graz
Institute of Sports Sciences
Mozartgasse 14
8010 Graz /Austria
E-Mail: peter.hofmann@uni-graz.at
+43 316 380 3903

Mag. Alexander Mueller
University of Graz
Institute of Sports Sciences
Mozartgasse 14
8010 Graz, Austria
E-Mail: alexander.mueller@uni-graz.at
+43 316 380 3903

Max L Eckstein, BSc
Swansea University
Applied Sport, Technology, Exercise and Medicine Research Centre (A-STEM)
A111 Engineering East, Fabian Way, Crymlyn Burrows
Swansea University, Swansea, SA1 8EN, UK
E-Mail: max.eckstein@swansea.ac.uk

Assoc. Prof. Dr. Richard M Bracken
Swansea University
Applied Sport, Technology, Exercise and Medicine Research Centre (A-STEM)
A111 Engineering East, Fabian Way, Crymlyn Burrows
Swansea University, Swansea, SA1 8EN, UK
E-Mail: r.m.bracken@swansea.ac.uk
Tel: +44 1792 513059

Signatures:

Assoc. Prof. Dr. Harald Sourij

Signature
Date

Dr. Othmar Moser

Signature
Date

Table of contents

1. Introduction 5
2. Research Question 6
3. Methods and Material 6
   3.1. Investigation Plan 6
   3.2. Study population 8
   3.3. Cardio-pulmonary Exercise (CPX) Test 9
   3.4. Pre-exercise Insulin Administration and Carbohydrate Intake Recommendations 9
   3.5. Measurements 10
   3.6. Primary Endpoint 10
   3.7. Secondary endpoints 10
4. Data Management and Analyses 11

Literature 12

1. *Introduction*

National and international Diabetes Associations endorse physical activity and regular exercise in people with type 1 diabetes (T1D) [1]. Exercise improves weight management, cardiovascular fitness, insulin sensitivity and wellbeing [2]. However, studies did not reveal a significant improvement in glycaemic control as measured by HbA_{1c} when patients were exercising regularly [3]. A reason for this finding might is the mismatch of insulin dose reduction and carbohydrate intake. Standard recommendations around exercise suggest pre-exercise bolus insulin reductions or even basal insulin dose reductions when patients are exercising more regularly [4][5][6]. Especially, early (>2 hrs) and excessive pre-exercise bolus insulin dose reductions may mask the beneficial effects of exercise on glycaemic control.

Little research exists on therapy strategies around high intensity exercises [4] or sports competitions in individuals with T1D [7]. During high-intensity exercise several physiological mechanisms underpin the complexity of therapy management in individuals with T1D: use of muscle glycogen and blood glucose as preferred fuel with minimal contributions from lipid and protein [8], increased concentrations of counterregulatory hormones like adrenaline, noradrenaline and cortisol [9] which might increase blood glucose concentration during exercise, increased risk of post-exercise hypoglycaemia due to higher absolute work rates and hepatic glycogen depletion etc. Precise strategies around prolonged high-intensity exercise are currently missing and might hinder people with T1D to compete in sports.

Therefore, the aim of this prospective study is:

- to investigate the effects of a running competition preparatory training on glycaemic control (HbA_{1c}, fructosamine, time spent in glycaemic ranges) in individuals with T1D
- to explore the changes in cardio-pulmonary markers following a running competition preparatory training and explore links to resultant running performance in individuals with T1D
- to characterise exogenous insulin and carbohydrate administration strategies that aim to increase the time spent in euglycemia during running competition
- to characterise the glycaemic variability in response to running a competition
- to characterise the physiological demands of running a marathon in people with T1D

2. Research Question

This prospective controlled study aims to investigate the effects of regular physical exercise in preparation for a road running competition on glycaemic control, time spent in glycaemic ranges and cardio-pulmonary markers

3. Methods and Material

3.1. Investigation Plan

The study will be performed in a prospective, non-interventional, observation design over a period of 10-12 weeks, consisting of 3 cardio-pulmonary exercise (CPX) tests, 3 venous HbA_{1c} and fructosamine measurements and a road running competition.

The Outpatient Clinics for Diabetes at the Medical University of Graz has accompanied and supported subjects with type 1 diabetes running at the Graz marathon over the last years already. This year subjects already planning to participate will participate in a structured and prospective observation study to record insulin treatment changes during the preparation phase, the marathon and the weeks thereafter.

After assessment of eligibility criteria, a comprehensive sports medical examination will be performed ahead of study inclusion to minimise the risk of exercise-induced adverse events. Participants will receive a written general training recommendation in preparation for the competition depending on individual capacities and aims. Additionally, detailed therapy strategies around exercise (insulin dose reductions, exercise carbohydrates, glycogen filling, hydration etc.) will be given to the participants. On the day of screening, patients will be fitted with a flash glucose monitoring system (if not already used) and insulin dosings, carbohydrates, exercise etc. will be documented digitally on the flash glucose monitoring system. Specific details on performed exercises (type, intensity, mode, duration) will be documented in an additional diary. Patients will receive a heart rate monitor which needs to be worn during the exercise sessions and at the day of competition (Fig1):

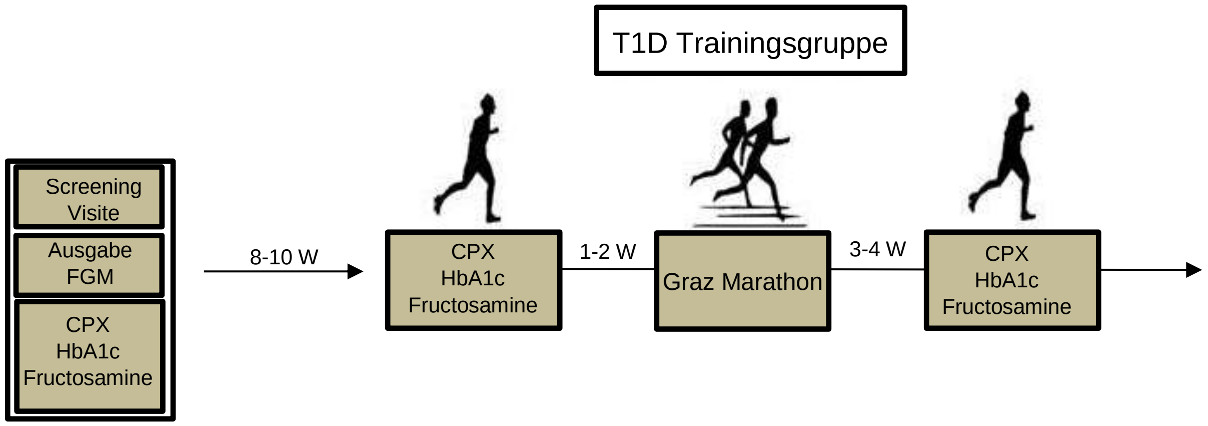

Figure 1: Study flow chart. FGM = flash glucose monitoring, CPX = cardio-pulmonary exercise test.

Over all, the study consists of 3 visits and a competition (the training group does not participate in the competition because of the study but participate in the study because they participate in the competition). The first visit includes the assessment of eligibility, written informed consent procedure, a medical examination, FGM fitting and the first CPX testing. At the second and third visit, participants will perform again a cardio-pulmonary exercise (CPX) test on the track to determine the first (VT1) and second ventilatory threshold (VT2) as well as the heart rate turn point (HRTP) [10][11].

Afterwards patients will be exercising regularly in preparation for the competition and document insulin therapy, carbohydrate intake and exercise frequency on their FGM as well as specific details around exercise in a diary. CPX tests will be repeated as given in figure 1. At the screening visit and CPX visits HbA_{1c} and fructosamine will be measured obtained from venous blood.

<u>Visit plan:</u>

1. Visit: Screening, informed consent, insertion of flash glucose monitoring (FGM) sensor, CPX test, measure of HbA_{1c} and fructosamine

2. Visit: CPX test, measure of HbA_{1c} and fructosamine

Running competition

3. Visit: CPX test, measure of HbA_{1c} and fructosamine

The exercise tests will be performed at the University of Graz (AUT). The Medical University of Graz (AUT) will recruit the participants and will also perform the medical examination. Diabetes specific markers will be analysed at the laboratory of the Medical University of Graz and at the sport science laboratory of the University of Graz (AUT).

3.2. Study population

Study participation is voluntarily after enlightenment by the study physician and given signed informed consent.

3.2.1. Inclusion Criteria

1) Type 1 Diabetes
2) Aged: 18 – 65 years (both inclusive)
3) A1c 5.5% - 10% (37 – 113 $mmol.mol^{-1}$) (both inclusive)
4) Multiple daily insulin injections (MDI) or insulin pump therapy (CSII)
5) Diabetes diagnosed > 12 months
6) BMI 18.5 – 30 kg/m^2 (both inclusive)
7) Planned participation at the Graz marathon

3.2.2. Exclusion Criteria

1) Severe Illness or disease, that confound the study results (assessed from the investigators)
2) Suspected allergy to trial products (FGM sensor)
3) Addiction to alcohol
4) Use of drugs
5) Mental incapacity

3.2.3. Subgroup

In addition to the subjects with type 1 diabetes, 10 healthy volunteers will participate in this trial. Therefore, placement of Freestyle Libre and application of ECG will be performed before the running competition. After finishing the running competition both devices will be removed by the study personnel.

3.3. Cardio-pulmonary Exercise (CPX) Test

Participants will perform a cardio-pulmonary exercise (CPX) test on the track until maximum exhaustion. At the beginning of the test, participants sit quietly on a chair (3 min) before they start to run with 6 km/h. Velocity increases then every 200 m by 0.5 km/h until maximum exhaustion.

Finally, 3 min active recovery at “walking speed” will be performed followed by 3 min sitting passive recovery. VT1, VT2, HRTP, maximum physiological markers and maximal velocity (V_{max}) will be used as markers of submaximal and maximal performance capacity.

3.4. Pre-exercise Insulin Administration and Carbohydrate Intake Recommendations

Participants will be informed by an information sheet to eat ≥ 5 - 7 (g CHO $.kg.^{-1}day^{-1}$) for the competition preparation period [12]. Insulin doses and carbohydrate amounts will be documented in the FGM system. Participants are told to apply their normal insulin doses or small reductions in bolus insulin (by 25%) during the intervention and document hypoglycaemic events (below 3.9 mmol/l) and additional eaten carbohydrates to counteract hypoglycaemia as well as hyperglycaemic episodes (>13.9 mmol/l) when they inject correction insulin. If hypoglycaemic episodes occurred frequently, additionally basal insulin can be reduced by up to 25%; however, basal insulin reductions need to be discussed in advance with the study physician.

For CPX testing and the individual exercise sessions, participants should consume the last pre-exercise meal at least 2 h before exercising to avoid drastic glucose decreases caused by bolus insulin injection. If the blood glucose is below 7 $mmol.l^{-1}$ immediately before the exercise, 15–30 g carbohydrates will be supplemented; in contrast to when the blood glucose is 10 $mmol.l^{-1}$ or above, carbohydrate feeding during exercise will be delayed until reaching 7 $mmol.l^{-1}$. When blood glucose decreases during exercise below 7 $mmol.l^{-1}$, again 15 g carbohydrates will be supplemented [12]. At each CPX visit, insulin therapy as well as the training concept will be discussed and in case recommendations will be individualised.

Before the competition, participants will reduce the last bolus insulin by 25-75% (when CSII basal rate will be reduced 90 min before the start of competition by 50-80%) depending on the distance they will be running (5 km, 10 km, 21 km or 42 km) [6]. Participants taking along 180 g carbohydrates (5 glucose gels) during the competition and consume further carbohydrates as often as needed to stay within a eu-glycaemic range. Afterwards carbohydrate intake will be calculated by means of consumed gels and additional carbohydrates.

3.5. Measurements

3.5.1. Measurements during CPX

Pulmonary gas exchange variables will be collected continuously during CPX testing by breath-by-breath measurement and averaged over 5 seconds. Heart rate will be measured continuously via chest belt telemetry during all tests and also averaged over 5 seconds. A 3-lead ECG will be obtained in all tests for cardiac monitoring. Capillary blood lactate and blood glucose taken from the earlobe will be measured at the rest, after the last exercise step at the end of the active recovery and finally, at the end of the passive recovery (Biosen S-line, EKF Diagnostics, GER) [9]. Additionally, glucose will be monitored via FGM and glucometer before the start of the CPX testing to avoid exercise-induced hypoglycaemia.

3.5.2. Measurement of Glycaemic Control

HbA_{1c} and Fructosamine will be collected from the antecubital vein at the before mentioned timepoints. Blood samples (15 ml) will be obtained from a cubital vein and analysed via ELISA (enzyme-linked immunosorbent assay) by the Medical University of Graz, AUT. Glycaemic ranges will be evaluated via interstitial glucose obtained from FGM during the training period and competition.

3.6. Primary Endpoint

- Difference in HbA_{1c} and Fructosamine from screening visit to last visit

3.7. Secondary endpoints

- Time spent in glycaemic ranges (hypoglycaemia <3.9 mmol/l, euglycaemia 3.9 mmol/l to 10 mmol/l, hyperglycaemia >10 mmol/l) from screening visit to pre-competition visit
- Numbers of hypoglycaemic episodes from screening visit to pre-competition visit
- Glycaemic variability from screening visit to pre-competition visit
- Difference in cardio-pulmonary variables assessed during CPX testing from screening visit to pre-competition visit
- Time spent in glycaemic ranges (hypoglycaemia <3.9 mmol/l, euglycaemia 3.9 mmol/l to 10 mmol/l, hyperglycaemia >10 mmol/l) during the competition depending on therapy adaptation
- Relationship of exogenous insulin and carbohydrate administration strategies and glycaemic variability during running competition
- Numbers of glycaemic episodes during the competition depending on therapy adaptation
- Glycaemic variability during the competition depending on therapy adaptation

- Relationship of competent performance and physiological demands

4. *Data Management and Analyses*

The documentation of data will be performed in a Case Report Form (CRF), FGM and diabetes diary according to Good Clinical Practice (GCP). After all measurements, data will be monitored before database lock. Descriptive statistic will be performed in both, intention to treat and per protocol participant dataset. A statistical analysis plan will be finished before database lock.

5. *Sample Size*

As we will collect data on subjects with type 1 diabetes participating in the Graz marathon competition and do not proactively recruit subjects for this non-interventional study, we will include approximately 6-10 subjects, depending on the number of people taking part in the marathon competition this year.

Literature

1. Colberg SR, Sigal RJ, Yardley JE, Riddell MC, Dunstan DW, Dempsey PC, et al. Physical Activity/Exercise and Diabetes: A Position Statement of the American Diabetes Association. Diabetes Care. 2016;39: 2065–2079. doi:10.2337/dc16-1728
2. Yardley JE, Hay J, Abou-Setta AM, Marks SD, McGavock J. A systematic review and meta-analysis of exercise interventions in adults with type 1 diabetes. Diabetes Res Clin Pract. Elsevier Ireland Ltd; 2014;106: 393–400. doi:10.1016/j.diabres.2014.09.038
3. Kennedy A, Nirantharakumar K, Chimen M, Pang TT, Hemming K, Andrews RC, et al. Does Exercise Improve Glycaemic Control in Type 1 Diabetes? A Systematic Review and Meta-Analysis. PLoS One. 2013;8. doi:10.1371/journal.pone.0058861
4. Moser O, Tschakert G, Mueller A, Groeschl W, Hofmann P, Pieber T, et al. Short-acting insulin reduction strategies for continuous cycle ergometer exercises in patients with type 1 diabetes mellitus. Asian J Sports Med. 2017;8. doi:10.5812/asjsm.42160
5. Campbell MD, Walker M, Bracken RM, Turner D, Stevenson EJ, Gonzalez JT, et al. Insulin therapy and dietary adjustments to normalize glycemia and prevent nocturnal hypoglycemia after evening exercise in type 1 diabetes: a randomized controlled trial. BMJ Open Diabetes Res Care. 2015;3: e000085–e000085. doi:10.1136/bmjdrc-2015-000085
6. Riddell MC, Gallen IW, Smart CE, Taplin CE, Adolfsson P, Lumb AN, et al. Exercise management in type 1 diabetes: a consensus statement. Lancet Diabetes Endocrinol. 2017;8587: 1–14. doi:10.1016/S2213-8587(17)30014-1
7. Buoite Stella A, Assaloni R, Tonutti L, Manca E, Tortul C, Candido R, et al. Strategies used by Patients with Type 1 Diabetes to Avoid Hypoglycemia in a 24×1-Hour Marathon: Comparison with the Amounts of Carbohydrates Estimated by a Customizable Algorithm. Can J Diabetes. 2017;41: 184–189. doi:10.1016/j.jcjd.2016.09.007
8. van Loon LJ, Greenhaff PL, Constantin-Teodosiu D, Saris WH, Wagenmakers AJ. The effects of increasing exercise intensity on muscle fuel utilisation in humans. J Physiol. 2001;536: 295–304. Available: http://www.ncbi.nlm.nih.gov/pubmed/11579177
9. Moser O, Tschakert G, Mueller A, Groeschl W, Pieber TR, Obermayer-Pietsch B, et al. Effects of high-intensity interval exercise versus moderate continuous exercise on glucose homeostasis and hormone response in patients with type 1 diabetes mellitus using novel ultra-long-acting insulin. Catapano A, editor. PLoS One. 2015;10: e0136489. doi:10.1371/journal.pone.0136489
10. Conconi F, Ferrari M, Ziglio PG, Droghetti P, Codeca L. Determination of the anaerobic threshold by a noninvasive field test in runners. J Appl Physiol. 1982;52: 869–73.
11. Hofmann P, Tschakert G. Special Needs to Prescribe Exercise Intensity for Scientific Studies. Cardiol Res Pract. 2011;2011: 1–10. doi:10.4061/2011/209302
12. Gallen IW, Hume C, Lumb A. Fuelling the athlete with type 1 diabetes. Diabetes, Obes Metab. 2011;13: 130–136. doi:10.1111/j.1463-1326.2010.01319.x
